# Over-expression of a retinol dehydrogenase (SRP35/DHRS7C) in skeletal muscle activates mTORC2, enhances glucose metabolism and muscle performance

**DOI:** 10.1038/s41598-017-18844-3

**Published:** 2018-01-12

**Authors:** Alexis Ruiz, Erez Dror, Christoph Handschin, Regula Furrer, Joaquin Perez-Schindler, Christoph Bachmann, Susan Treves, Francesco Zorzato

**Affiliations:** 1 Departments of Anesthesia and of Biomedicine, Basel University Hospital, Hebelstrasse 20, 4031 Basel, Switzerland; 2 Max Planck Institute of Immunobiology and Epigenetics, 79108 Freiburg, Germany; 3 Biozentrum, University of Basel, CH-4056 Basel, Switzerland; 4 Department of Life Sciences, General Pathology section, University of Ferrara, Via Borsari 46, 44100 Ferrara, Italy

## Abstract

SRP-35 is a short-chain dehydrogenase/reductase belonging to the DHRS7C dehydrogenase/ reductase family 7. Here we show that its over-expression in mouse skeletal muscles induces enhanced muscle performance *in vivo*, which is not related to alterations in excitation-contraction coupling but rather linked to enhanced glucose metabolism. Over-expression of SRP-35 causes increased phosphorylation of Akt_S473_, triggering plasmalemmal targeting of GLUT4 and higher glucose uptake into muscles. SRP-35 signaling involves RARα and RARγ (non-genomic effect), PI3K and mTORC2. We also demonstrate that all-trans retinoic acid, a downstream product of the enzymatic activity of SRP-35, mimics the effect of SRP-35 in skeletal muscle, inducing a synergistic effect with insulin on AKT_S473_ phosphorylation. These results indicate that SRP-35 affects skeletal muscle metabolism and may represent an important target for the treatment of metabolic diseases.

## Introduction

Skeletal muscle is the largest body organ comprising approximately 40% of total body weight under normal conditions, it is not only important for movement and posture but also for metabolism and thermogenesis^1,2^. Furthermore, skeletal muscle adapts to different environmental conditions and load requirements by modifying its fiber type composition and glycolytic or oxidative characteristics^3,4^. Muscle fiber type composition is determined mainly by the expression of specific myosin heavy chain isoforms: MyHC I in slow fibres, MyHC type IIa in fast oxidative fibres and MyHC IIx/b in fast glycolytic fibres^4,5^. The differences in contractile properties between slow and fast fibers also depends on the higher density of sarco/endoplasmic reticulum Ca^2+^-ATPase (SERCA), expression of Ca^2+^- binding proteins such as parvalbumin, calsequestrin 1 and calsequestrin 2, and a higher mitochondrial content in slow type I fibers compared to fast type II fibers^4,6,7^.

Skeletal muscle is responsible for 70–75% of the insulin stimulated glucose uptake;^8^ part of the energy obtained from glucose is used to fuel muscles and the remaining is stored as glycogen^9^. Two glucose transporters are expressed in skeletal muscle: GLUT4 and GLUT1 which are insulin-sensitive and insulin-insensitive, respectively^10–12^. Insulin receptor stimulation activates a number of downstream signaling proteins including phosphoinositide 3-kinase (PI3K) and protein kinase B (PKB or Akt) which induce GLUT4 translocation to the plasma membrane where it facilitates blood glucose clearance and glucose uptake into muscles^9,13^.

In the past years our laboratory has discovered novel proteins of the skeletal muscle sarcoplasmic reticulum (SR)^14^. One such protein we identified at the molecular level is SRP-35, a 35 kDa membrane bound protein enriched in sarcotubular membranes. The NH_2_-terminus of SR-P35 encompasses a short hydrophobic sequence associated to SR membranes, whereas its C-terminal domain faces the myoplasm. Sequence comparison and functional *in vitro* experiments established that SRP-35 is a short-chain dehydrogenase/ reductase belonging to the DHRS7C [dehydrogenase/reductase (short-chain dehydrogenase/reductase family) member 7C] subfamily^15,16^, using retinol (Vit. A) as its substrate and leading to the formation of *all*-trans-retinaldehyde^17^. The latter is the substrate for an irreversible oxidative reaction generating *all*- trans retinoic acid (atRA). Retinoic acid (RA) acts as a ligand for the family of nuclear retinoic acid receptors (RAR) and nuclear retinoid X receptors (RXR), encoded by separate genes for the three subtypes, α, β and γ of both RAR and RXR in mammalian cells^18^. Nuclear retinoic acid receptors regulate the expression of genes involved in a variety of biological processes ranging from tissue development to cell differentiation^19^. In addition, RA and its nuclear receptors exert non-genomic extra-nuclear activity, further extending the spectrum of effects of RA on biological functions^20^. Indeed, in the past few years a number of studies have revealed that RA influences cell metabolism^21,22^, by modulating, the (i) phosphorylation state of AMPK and Akt^23^ and (ii) rate of glucose uptake by L6 myotubes^24^.

Because of the important metabolic role of RA, we generated a transgenic mouse line overexpressing SRP-35 in skeletal muscle. Our results show that these transgenic mice (SRP35TG) exhibit enhanced running capacity, enhanced glucose uptake leading to larger glycogen stores. Furthermore, the presence of the transgene induces a stronger activation of PI3K and of mammalian target of rapamycin complex 2 (mTORC2) signaling pathways, triggering the phosphorylation of Akt_S473_, which promote GLUT4 translocation onto the plasma membrane. The metabolic changes observed in SRP35TG mice could be mimicked by treating skeletal muscles of wild type mice with atRA.

## Results

### Impact of SRP-35 over-expression on the expression of SR ECC proteins and on muscle function

**Figure 1:**
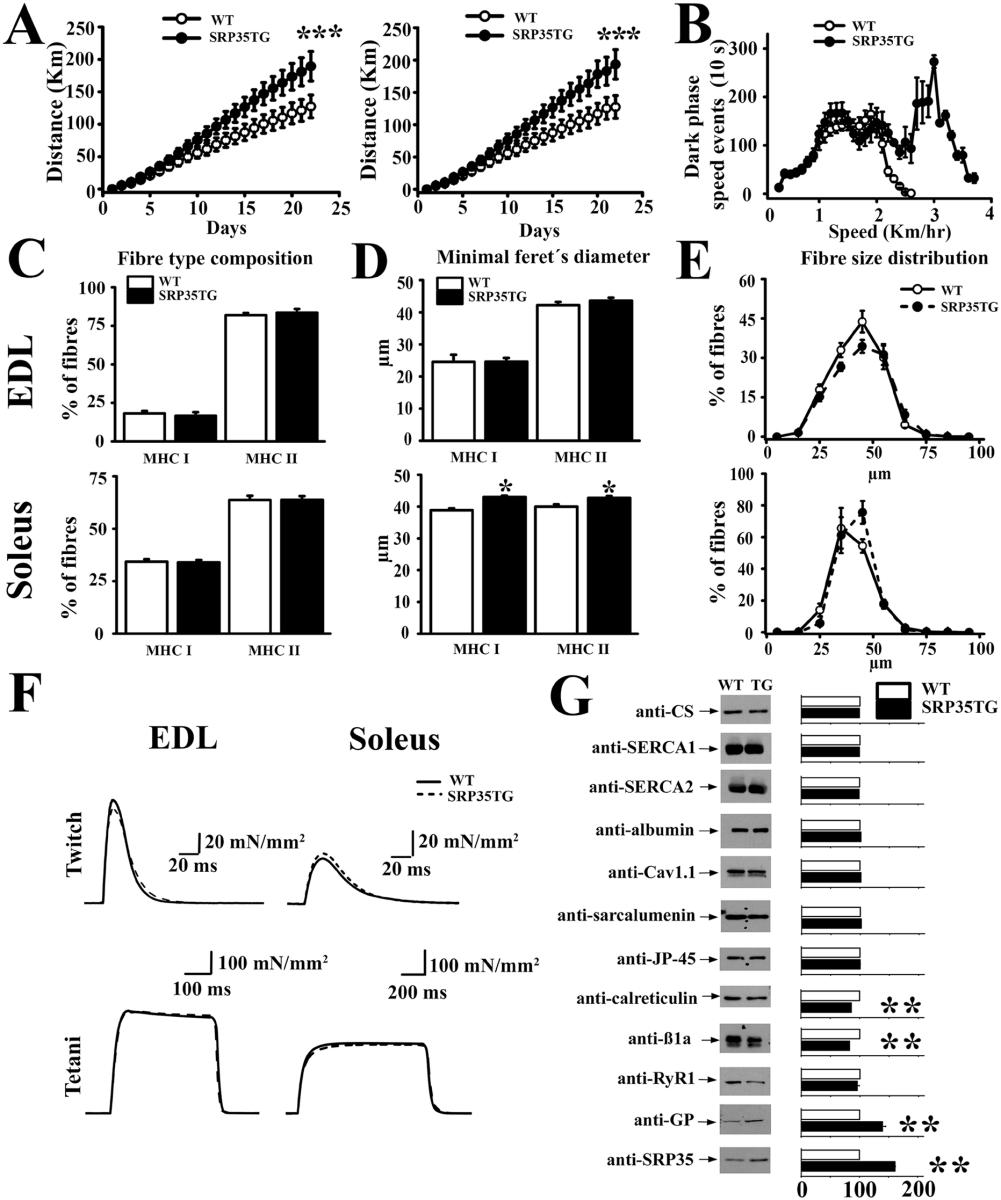
Spontaneous activity, fiber type composition, force measurements and SR protein composition of muscles from SRP35TG mice. (**A)** Spontaneous daily activity of WT (white symbols) and SRP35TG (black symbols) mice. Data points from 7 months (left) and 14 months (right) old mice are expressed as mean ± S.D., n = 16, ***p < 0.001 Mann–Whitney test. **(B)** Dark phase speed events recorded from the 15^th^ to 20^th^ day of running (mean ± S.D., n = 16). **(C)** and **(D**) Fibre type composition Minimal Feret’s diameter determined by myosin-heavy chain (MyHC) immunohistochemistry in EDL and soleus muscles of slow (MyHCI) and fast (MyHCII) fibres (mean ± S.D., n = 10, *p < 0.05 Student’s *t* test). (**E)** Fibre size distribution of EDL and soleus muscles from WT and SRP35TG mice (mean ± S.D., n = 10). **(F**) Mechanical properties of EDL and soleus muscles from WT (continuous line, representative trace of experiments carried out in 7 mice) and SRP35TG (dashed line, representative trace of experiments carried out in 13 mice) mice. Twitch force stimulated by a 15 V pulse of 0.5ms duration. Maximal tetanic force induced by a train of pulses delivered at 200 and 150 Hz for EDL and soleus, respectively. (**G)** Western blot analysis. Thirty-five µg of total SR protein were loaded per lane, separated on 6% or 10% SDS/PAGE. Bar histograms represent the mean ± S.D. intensity of the immunoreactive band in SRP35TG fraction expressed as % of the intensity of the band in WT mice (n = 6, **p < 0.01 Student’s *t* test).

We generated TG mice encoding mouse SRP-35 under the skeletal muscle specific creatine kinase promoter (Supplementary Fig. 1A). The presence of the transgene was confirmed by polymerase chain reaction (PCR) (Supplementary Fig. 1B), Coomassie Blue staining on a total SR extract of skeletal muscle from WT and SRP35TG mice (Supplementary Fig. 1C asterisk) and Western blotting. We obtained three mouse lines expressing the TG to different extents: line 4, 5 and 6 over-expressing SRP-35 by 28%, 61% and 23%, respectively (Supplementary Fig. 1D). In this study, we performed most of the experiments on muscles from line 5 and their respective wild-type (WT) littermates but similar results were obtained on the other TG lines. We first investigated *in vivo* and *in vitro* muscle function using the voluntary running wheel and electrically stimulated force generation, respectively. Figure 1 shows that after 21 days young (7 months, Fig. 1A left panel) and old (14 months, Fig. 1A right panel) SRP35TG male mice (closed circles) ran approximately 50 Km more (n = 16 p < 0.001, Mann-Whitney test) compared to their age matched WT littermates (Fig. 1A, open circles). The higher running capacity of the SRP35TG mice between day 15 and day 20 is also paralleled by a remarkable increase in the number of 10 s high speed events (>2.5 km/hr) occurring during the dark phase (Fig. 1B). The enhanced running capacity is not linked to changes in fiber type composition since we found an equal distribution of MyHC I and MyHC II positive fibers in *extensor digitorum longus* (EDL) and soleus muscles in WT and SRP35TG mice (Fig. 1C). However, over-expression of SRP-35 caused a small (10%) but significant (p < 0.05 Student’s *t* test) increase of the minimal Feret’s diameter of MyHC type I and MyHC type II fibers (Fig. 1D and E) in soleus. The mechanical properties of WT and SRP35TG mice are similar (Fig. 1F and Supplementary Table 1) indicating that the enhanced running is not due to a gain of function of the excitation-contraction coupling (ECC) mechanism. This conclusion is also consistent with the absence of changes in the content of RyR1, Ca_v_1.1 and calsequestrin, the main proteins constituting the ECC macromolecular complex (Fig. 1G). On the other hand, SRP-35 over-expression was accompanied by a significant reduction of the ER(SR) calcium binding protein calreticulin (n = 6, p < 0.01, Student’s *t* test) and of the β1a subunit of the dihydropyridine receptor (n = 6 p < 0.01, Student’s *t* test). SRP35TG muscles also display a 40% increase of glycogen phosphorylase content (GP; Fig. 1G; n = 6 p < 0.01, Student’s t test), a key enzyme involved in glucose metabolism activation during muscle contraction^25^. Because of this increase of glycogen phosphorylase we hypothesized that the enhanced running capacity was the result of an effect on glucose metabolism.

### SRP35TG mice show enhanced glucose metabolism

**Figure 2:**
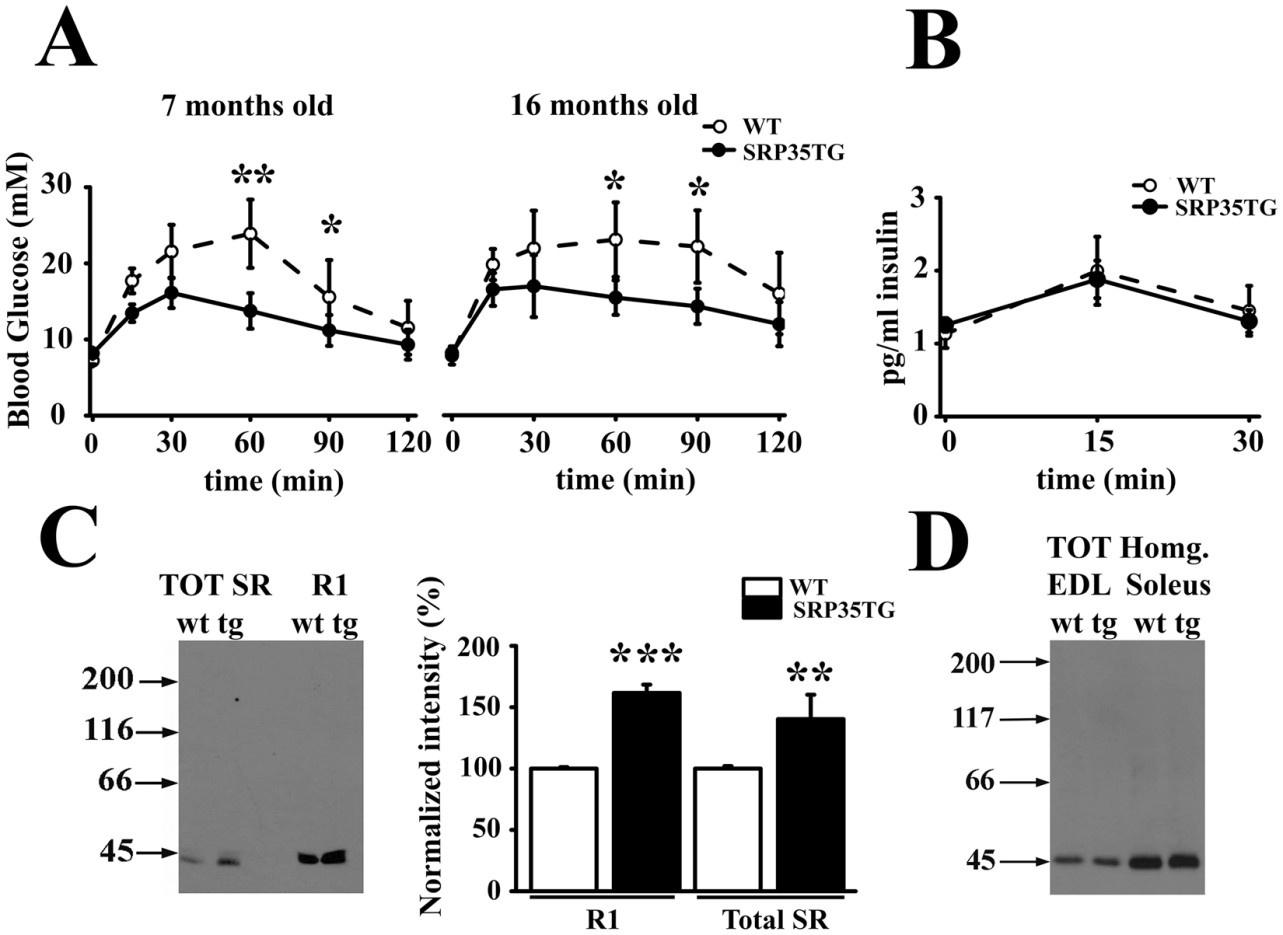
Glucose uptake and plasma membrane GLUT4 levels are increased in SRP35TG mice. **(A)** Glucose tolerance test (ipGTT) in WT (n = 8) and SRP35TG (n = 8) mice, values are expressed as mean ± S.D. blood glucose levels; *p < 0.05; **p < 0.01 Student’s *t* test. (**B)** Circulating insulin in WT (n = 8) and SRP35TG (n = 8), is expressed as mean ± S.D. pg of insulin measured after glucose injection. Values were not significantly different in WT and SRP35TG mice (Student’s *t* test). **(C)** GLUT4 content of total SR and R1 fraction (tubule T/sarcolemma membranes) of skeletal muscles from wild type (wt; n = 8) and SRP3TG (tg; n = 8) mice. Ten µg of protein were loaded per lane and separated on 7.5% SDS/PAGE (left); bar histograms of GLUT4 content in skeletal muscles from SRP35TG (black bars), normalized to control levels (white bars). Results are expressed as mean ± S.D., **p < 0.01; ***p < 0.001 Student’s *t* test. **(D)** Western blot of GLUT4 in total homogenate from EDL and soleus muscles.

*In vivo* glucose clearance was performed by the intraperitoneal glucose tolerance test (ipGTT) in young and old (7 and 16 months old, respectively) mice. Both age groups of SRP35TG mice showed a two-fold reduction in serum glucose levels 60 min after the glucose challenge compared to WT mice (Fig. 2A; n = 8 p < 0.05, Student’s *t* test). Additionally, there was a significant difference in glucose clearing kinetics between young and old WT mice at 90 and 120 min. Interestingly in SRP35TG mice the glucose clearance curves of young and old mice were similar, with no delay in clearance, suggesting a protective effect of SRP-35 over-expression in age-related glucose uptake processes^26^. The enhanced glucose clearance was not related to altered levels of insulin release, as blood insulin levels were similar in WT and SRP35TG mice (Fig. 2B).

Skeletal muscles express two main types of glucose transporters, GLUT1 which is insulin insensitive and GLUT4 which is exocytosed onto the plasma membrane in response to insulin. GLUT1 expression levels in total muscle homogenates and in the R1 fraction enriched in transverse tubules and plasma membrane^27^, was similar in WT and SRP35TG mice (Supplementary Fig. 2A and B). On the other hand GLUT4 expression in total SR and R1 fraction from SRP35TG mice was approximately 50% higher (n = 8  p < 0.01, Student’s *t* test) compared to WT mice (Fig. 2C). The total amount of GLUT4 in the total muscle homogenates from both EDL and soleus was unchanged (Fig. 2D), indicating that SRP35TG mice constitutively activate or activate more efficiently the signal(s) responsible for GLUT4 translocation onto the plasma membrane and that the increased membrane expression of GLUT4 is not caused by enhanced synthesis of this transporter.

**Figure 3:**
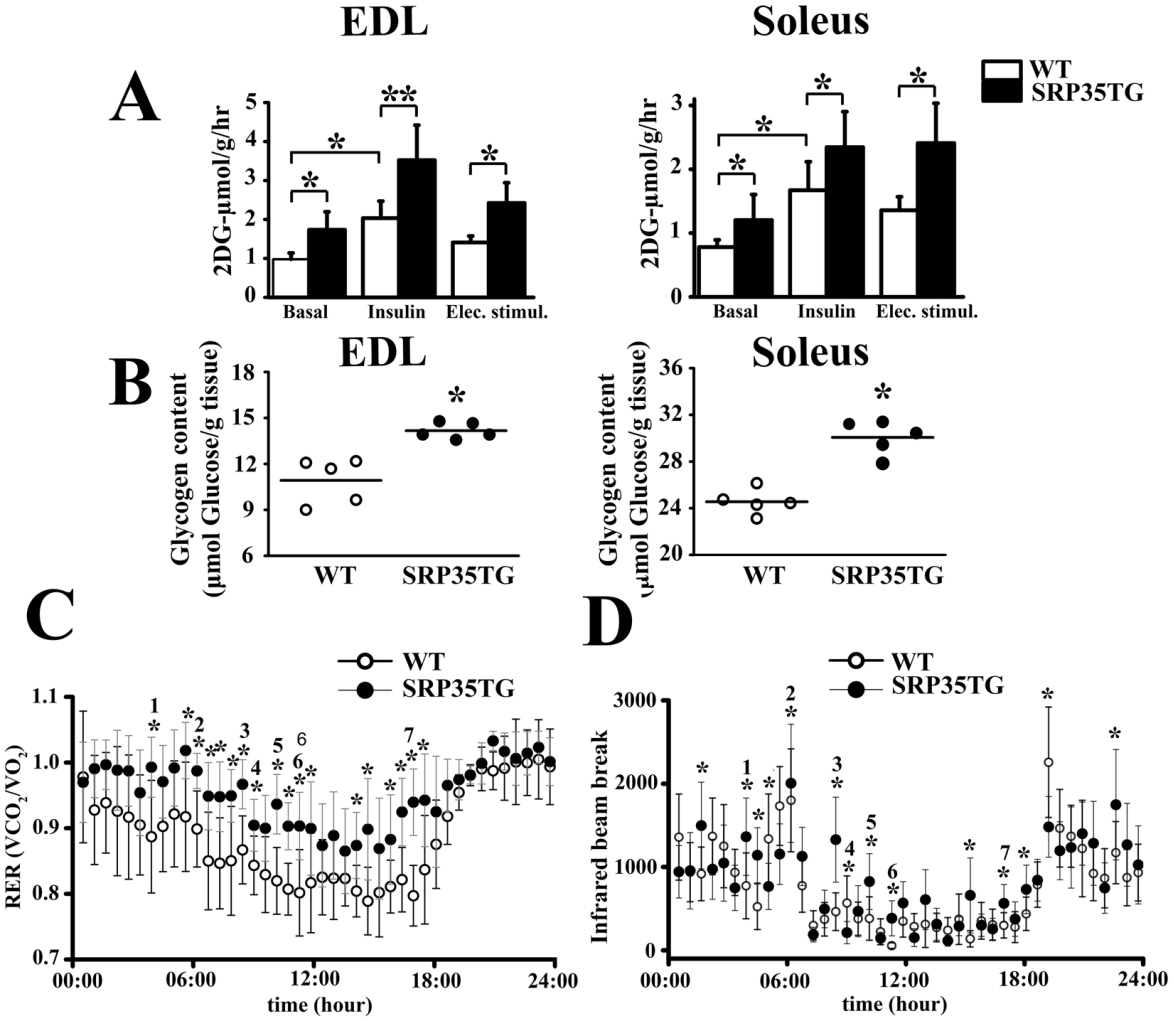
Skeletal muscle glycogen stores and RER in SRP35TG mice. **(A)** *In vitro* glucose uptake in EDL and soleus muscles from WT and SRP35TG. Three conditions were used: (i) no treatment (Basal; n = 8); (ii) 10 min incubation with 100 nM insulin (n = 6), (iii) electrical stimulation with a train of tetani (80 Hz, 300 ms duration) delivered at 0.27 Hz for 5 min (n = 6). Data is expressed as mean ± S.D.; *p < 0.05; **p < 0.01 Student’s *t* test. **(B)** Glycogen content was assessed enzymatically in total homogenates from EDL and soleus muscles. Each symbol represents the mean value of both muscles of a single mouse; the median value is represented by the horizontal black line; *p < 0.05 Student’s *t* test. **(C)** Respiratory exchange ratio (RER) was measured in CLAMS cages in WT (n = 8) and SRP35TG (n = 8) mice, fed a standard chow diet. Values are expressed as mean ± S.D.; *p < 0.05 Student’s *t* test. **(D)** Spontaneous locomotor activity was recorded on X, Y and Z axis, and is expressed as mean ± S.D.; n = 8, *p < 0.05 Student’s *t* test.

The increased running capacity of SRP35TG mice may thus be related to the enhanced capacity of their skeletal muscles to remove circulating glucose and store it. We verified this directly (i) by monitoring the capacity of isolated muscles to take up glucose and (ii) by monitoring muscle glycogen stores. EDL and soleus muscles were assessed for their capacity to take up 2-deoxy-D-glucose-[^3^H]) by three different protocols, namely (i) no stimulation, (ii) after challenge with insulin (100 nM for 10 min), and (iii) by a train of action potentials delivered at 80 Hz for 5 min (Fig. 3A). Under basal conditions, 2-DG-[^3^H] uptake in muscles from SRP35TG (7 months old) male mice was significantly increased compared to WT (Fig. 3A, n = 8; p < 0.05 Student’s *t* test). This difference in glucose uptake was further increased either by insulin treatment or electrical stimulation. Insulin promoted more glucose uptake by EDL and soleus muscles of SRP35TG mice (Fig. 3A filled bars, n = 6; p < 0.05 Student’s *t* test) compared to WT littermates (Fig. 3A, empty bars). Muscles from SRP3TG mice accumulated more 2DG-[^3^H] compared to those from WT littermates also after electrical field stimulation (Fig. 3A, n = 6; p < 0.05 unpaired *t* test). Altogether our data demonstrates that both fast and slow muscles isolated from SRP35TG mice show significant higher glucose uptake compared to WT littermates, and that the highest increase in glucose uptake was observed after insulin stimulation. Glucose taken up by muscles is stored as glycogen, a glucose polymer acting as a readily available energy store for muscle activity. Thus the higher glucose uptake by SRP35TG muscles should result in larger glycogen stores and this was directly verified by assessing glycogen content by an enzyme-coupled assay. Figure 3B shows that the glycogen content in muscles from 7 months old SRP35TG male mice was around 30% higher compared to that in WT littermates (n = 5; p < 0.05 Student’s *t* test). Since muscle glycogen content is an important factor determining the respiratory exchange rate (RER), we also assessed basic metabolism using a CLAMS apparatus. Figure 3C shows cumulative RER of WT (empty circles) and SRP35TG (filled circles) littermates. Each data point represents the average of RER from 7 months old male mice and shows a high degree of variability. There were no significant differences in the RER during the dark phase. However, 10 out 23 RER data points of the light phase acquired on the last day of measurements were significantly increased in SRP35TG mice compared to WT (n = 8; p < 0.05 Student’s *t* test). These events likely result from an increase of voluntary motor activity because we observed a parallel significant increase of the infrared beam breaks data points (Fig. 3D).

### Overexpression of SRP-35 affects Akt and AMPK phosphorylation

**Figure 4:**
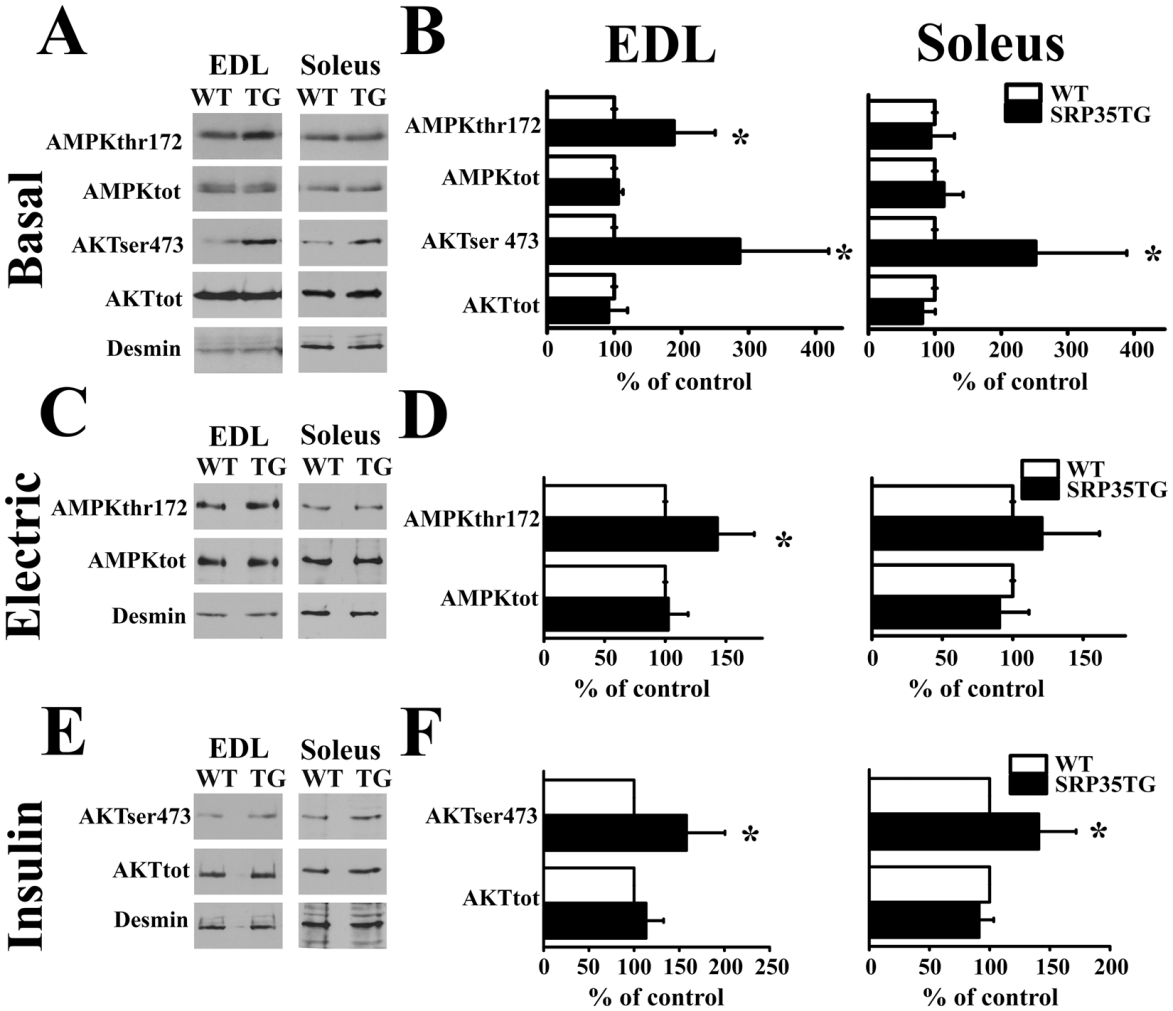
AMPK and Akt phosphorylation levels are increased in muscles from SRP35TG mice. Western blots of total homogenates from EDL and soleus muscles. Phosphorylation of AMPK_T172_ and Akt_S473_ was determined under the following experimental conditions. **(A)** and (**B**) basal conditions (n = 7). **(C)** and **(D)** electrical stimulation with a train of tetani (80 Hz, 300 ms duration) delivered at 0.27 Hz for 5 min (n = 6). **(E**) and (**F**) stimulation with 100 nM insulin (n = 6). Representatives western blots from the three conditions are show in panels (A, C and E) and the Bar histograms in panels (B, D and F). Fifty µg of protein from total muscle homogenates were loaded per lane, separated on 10% SDS-PAGE and blotted onto nitrocellulose. The immunoreactivity to desmin was used to normalize protein loading. Data are presented as % ± S.D. of WT values (control); *p < 0.05 Student’s *t* test.

GLUT4 membrane translocation and glucose uptake in skeletal muscle result from the activation of two major pathways, namely Akt and AMP-activated protein kinase (AMPK). Akt activation is linked to the stimulation of the insulin receptor, while AMPK activation may occur via multiple mechanisms, including muscle contraction^28^. To assess the involvement of these signaling pathways we performed quantitative western blot analysis and determined the degree of phosphorylation of Akt (Ser473 and Thr308) and AMPK (Thr172) in total muscle homogenates. Our results show that in muscles from SRP35TG and WT (7 months old) male mice the absolute content of Akt and AMPK in EDL and soleus at rest, after electrical stimulation or after the addition of insulin are similar (Fig. 4). Under resting conditions in soleus muscles the level of phosphorylation of AMPK_Thr172_ was similar in SRP35TG and WT littermates (Fig. 4A and B), whereas EDLs from SRP35TG show a significant increase (n = 6; p < 0.05, Student’s t test) of AMPK_T172_ phosphorylation compared to WT littermates (Fig. 4A and B). Upon delivery of a repetitive train of action potentials the phosphorylation level of AMPK_T172_ in EDL from SRP35TG mice was further increased from resting levels by 49.3% (Fig. 4C and D, p < 0.05 Student’s *t* test), whereas in soleus the increase was not statistically significant (Fig. 4C and D). As to the phosphorylation level of Akt, we found different effects concerning Thr 308 and Ser 473, namely overexpression of SRP-35 only increases the phosphorylation of Ser 473 in both EDL and soleus (results for Akt_t308_ are not shown). Under resting conditions we observed an increase of the Akt_S473_ phosphorylation level in EDL and in soleus, respectively (286.1 ± 132% and 251 ± 137%, mean ± S.D., n = 7, p < 0.05, Student’s t test; Fig. 4A and B). Insulin caused a further increase of the resting phosphorylation level of Akt_S473_ in EDL and soleus muscles by 58 and 40%, respectively (n = 7, p < 0.05, Student’s t test Fig. 4E and F).

### atRA causes Akt_S473_ phosphorylation

**Figure 5:**
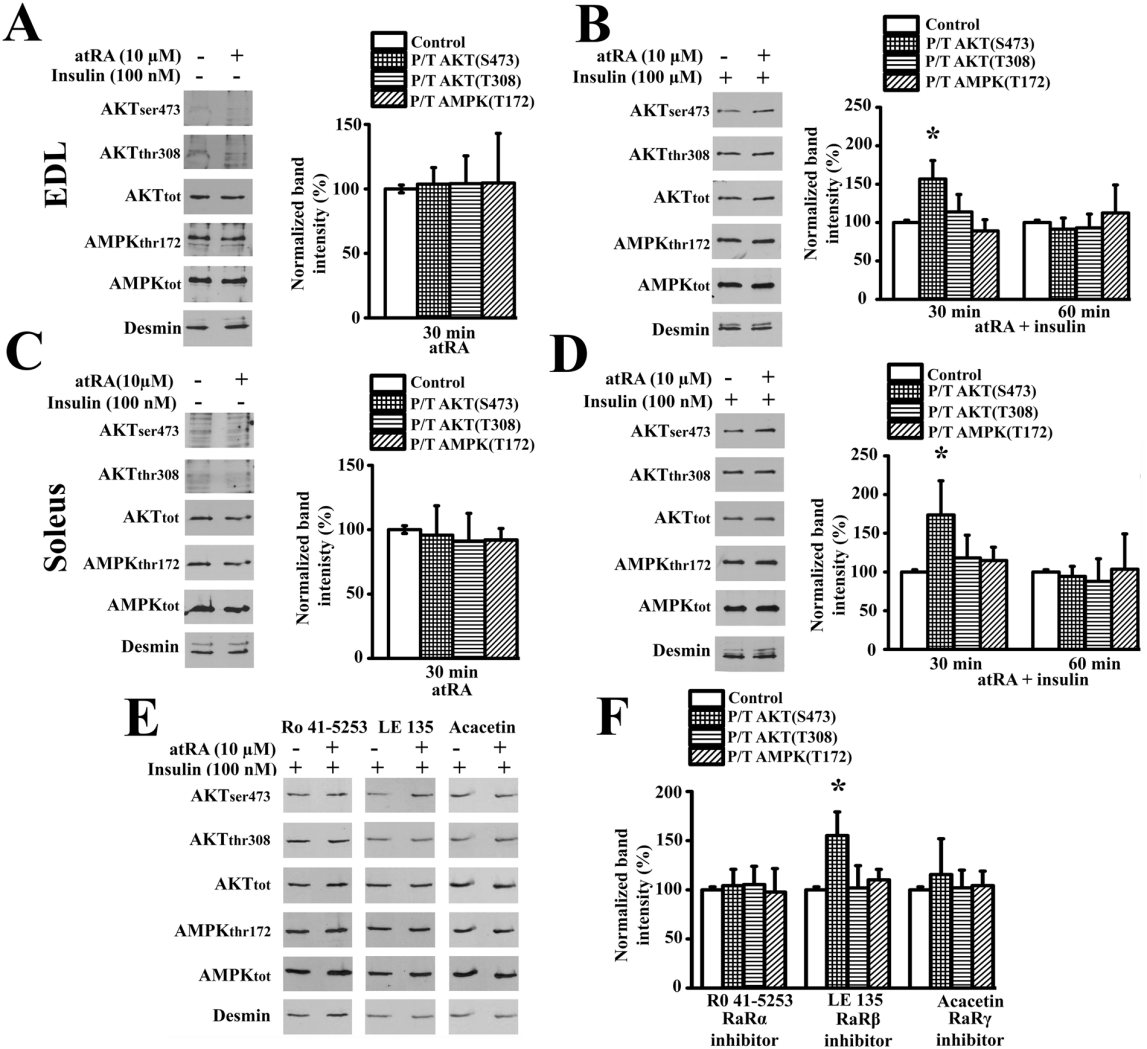
atRA activates AktS473 phosphorylation in WT EDL and soleus muscles. Fifty µg of protein from total muscle homogenates were loaded per lane, separated on 10% SDS-PAGE and blotted onto nitrocellulose. AMPK_T172_, Akt_S473_ and Akt_T308_ phosphorylation levels were measured by western blotting on total homogenates from EDL and soleus with specific anti-phospho Ab; P/T = Phosphorylated protein/Total protein. The immunoreactivity to desmin was used to normalize gel loading. Data are presented as % ( ± S.D.) of control (empty bar); * p < 0.05 Student’s *t* test. **(A)** and **(C)** Skeletal muscles isolated from WT mice were incubated with 10 μM all-*trans*-retinoic acid (atRA, bars; n = 6). Left side: representative western blots at 30 min of incubation; right side: bar histogram plots. Control values (empty bar) for normalization were obtained by incubating the contralateral muscle in the presence of the vehicle solution (DMSO). **(B)** and **(D)** EDL and soleus muscles incubated with 100 nM insulin plus or minus 10 μM atRA. Left side: representative western blots at 30 min of incubation; right side: bar histogram plots (n = 6 *p < 0.05 Student’s *t* test). Control values (empty bar) for normalization were obtained by performing the experiments in the presence of 100 nM insulin plus vehicle (DMSO). **(E)** and **(F)** EDL muscles were incubated for 30 min in presence of 100 nM insulin, RARα, RARβ and RARγ inhibitors plus or minus 10 μM atRA. Control values (empty bar) were obtained in the presence of 100 nM insulin, plus the RAR inhibitor. Data are presented as mean ± S.D., n = 6, *p < 0.05 Student’s *t* test.

Although the exact mechanism by which SRP-35 affects Akt phosphorylation is not know, it might be due to the formation of RA resulting from the oxidation of *all*-trans-retinaldehyde, the precursor of RA generated by the up-regulation of the SRP-35 dehydrogenase activity^17^ in muscles of SRP35TG mice. We tested this by assessing whether the phosphorylation of Akt_S473_ could be mimicked by treatment of muscles from WT mice with pharmacological concentrations of atRA^29^. Treatment of intact EDL and soleus muscles for 30 and 60 min with 10 μM atRA alone did not cause significant changes in the total amount of Akt and AMPK protein content (see Supplementary Fig. 2C) and had no effect on the phosphorylation status of Akt_S473_, Akt_T308_, and AMPK_T172_ (Fig. 5A and C). However, atRA may synergize with activators of the Akt and AMPK signaling pathways^30^. Indeed incubation of intact EDL and soleus muscles with atRA in the presence of insulin significantly stimulates the phosphorylation of Akt_S473_ by 166.6 ± 24% and 173.6 ± 44%, respectively (mean ± S.D., n = 6, p < 0.05, Student’s *t* test Fig. 5B and D). This increase occurred after 30 min and subsequently declined to control values by 60 min. The effect of atRA in the presence of insulin was specific for Akt_S473_ since we did not observed an increase in the phosphorylation of either Akt_T308_ or AMPK_T172_ (Fig. 5B and D). The fast time course of atRA-induced Akt_S473_ is consistent with a non-genomic action and this was further investigated by testing the effect of Retinoic acid Receptor α, β and γ inhibitors on Akt_S473_ phosphorylation. Figure 5E and F show that phosphorylation of Akt_S473_ induced by atRA in the presence of insulin was abolished by RARα and RARγ inhibitors and was insensitive to the RARβ inhibitor

### atRA stimulation of Akt phosphorylation is mediated by mTOR complex 2

**Figure 6:**
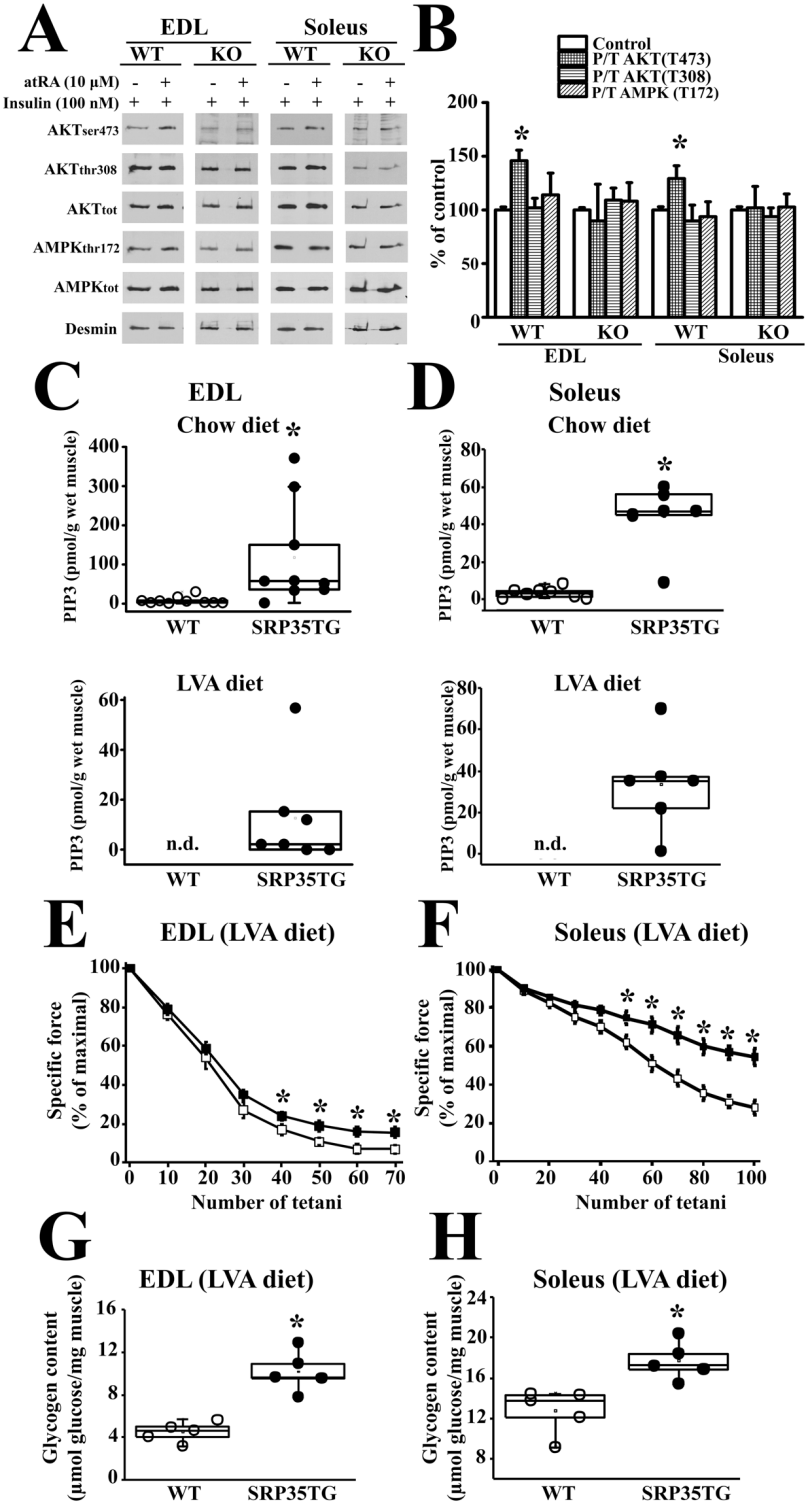
SRP-35 and atRA activation of Akt_S473_ is controlled by mTORC2 and the PI3K signaling pathway. **(A)** and **(B)** Western blot images and bar histograms showing the phosphorylation of Akt and AMPK in EDL and soleus muscles from WT (n = 5) and RimKO (n = 5) mice incubated with or without 10 μM atRA in presence of 100 nM insulin. Fifty µg of total homogenate protein were loaded per lane, separated on 10% SDS/PAGE and blotted onto nitrocellulose. P/T = Phosphorylated protein/Total protein. Anti-desmin immunoreactivity was used as loading control (bars represent the mean ± S.D., *p < 0.05 Student’s *t* test). **(C)** PIP_3_ levels from EDL muscles isolated from WT and SRP35TG mice fed a standard chow diet (Top panel) and on a low vitamin A diet (LVA, 4 I.U/Kg, Bottom panel). Each symbol represents PIP_3_ values from a single mouse; the median value of the data is shown in the box-plot; n.d. = non detectable (muscles from 7 mice) *p < 0.05 Mann-Whitney test. **(D)** PIP_3_ levels from soleus muscles isolated from WT and SRP35TG mice fed a standard chow diet (Top panel) and on a low vitamin A diet (LVA, 4 I.U/Kg, Bottom panel). Each symbol represents PIP_3_ values from a single mouse; the median value of the data is shown in the box-plot; *p < 0.05 Mann -Whitney test. **(E)** and **(F)** EDL (**E**) and Soleus (**F**) muscles from WT (open squares) and SRP35TG (closed squares) mice kept for 2 generations under low Vit A diet (LVA), were stimulated in the presence of 100 nM insulin with a train of tetanic stimulation (EDL: 70 Hz, 300 ms duration; Soleus 50 Hz, 600 ms duration) delivered at 0.33 Hz. Maximal specific force (mN/mm^2^) of the first tetanic contracture is expressed as 100%. Each point represents the mean ± S.D.; n = 5 WT and 5 SRP35TG; *p < 0.05 Mann-Whitney. **(G)** and **(H)** Glycogen content was assessed enzymatically in total homogenates from EDL and Soleus muscles at the end of the fatigue protocol. Each symbol represents the value from a single mouse; the median value of the data is shown in the box-plot; *p < 0.05 Mann-Whitney test.

The higher extent of phosphorylation of Akt_S473_ in SRP35TG mice and the *in vitro* experiments with atRA may result from the activation of the Rictor/mTORC2 complex^31–33^. In order to verify this we performed control experiments on muscles isolated from muscle specific Rictor knock-out (RImKO) mice^34^, a mouse model exhibiting down-regulation of the mTORC2. EDL and soleus muscles from (3 months old) male RImKO mice were treated with 100 nM insulin in the presence or absence of 10 μM atRA. Under these conditions the RImKO mice failed to show significant changes in Akt_S473_ phosphorylation (Fig. 6A and B). This effect is specific since under identical experimental conditions phosphorylation of Akt_S473_ occurred in EDL and soleus muscles from WT littermates.

### PIP_3_ content in total muscle homogenates from WT and SRP35TG mice

The data obtained with RImKO mice demonstrate that atRA synergizes with physiological activators in triggering the enzymatic activity of mTORC2. PIP_3_, a product of the PI3K signaling pathway, is a physiological activator of the kinase activity of mTORC2^35,36^. We next measured PIP_3_ content in EDL and soleus muscles from SRP35TG and WT mice. Figure 6 (panels C and D) shows that both EDL and soleus muscles from (4 months old) male SRP35TG mice have a significant increase of PIP_3_ content (p < 0.05, Mann-Whitney test). The intracellular level of atRA is influenced by the content of Vitamin A in the diet and a reduction of Vitamin A from 14 to 4 IU/g in the mouse chow was reported to induce a decrease in atRA content in various mouse tissues^37^. We assume that at any given intracellular retinol concentration the muscle fibres over-expressing SRP-35 would generate greater amounts of *all*-trans-retinaldehyde the precursor of the irreversible oxidation reaction leading to atRA formation. Thus one would expect that if mice were fed a low Vitamin A diet, the SRP35TG mice would generate more atRA compared to WT littermates and that the higher atRA levels would be associated with enhanced PI3K activity. We found that in (4 months old) male mice that had been fed for two generations with a low vitamin A (4 IU/g) diet, the PIP_3_ content in EDL and soleus muscles from WT mice under resting conditions was below the detection limit of the method, whereas in EDL muscles from SRP35TG mice the level is approximately 10% of that measured in EDL muscles of mice fed a standard chow (Fig. 6C lower panel) and in soleus muscles the PIP_3_ level is approximately 70% of that measured in soleus muscles of mice fed a standard chow diet. The higher PI3K activity in muscles from SRP35TG kept on a low vitamin A diet should enhance mTORC2/Akt signaling which in turn may lead to higher glucose uptake and increased glycogen stores. If this were the case, one would expect better muscle performance. We tested this possibility by investigating the *in vitro* resistance to fatigue in EDL and Soleus isolated from mice kept on a low vitamin A diet for two generations, by measuring the force developed after a train of tetanic stimulations of EDL (70 Hz, 300 msec duration) and soleus muscles (50 Hz, 600 msec duration) delivered at 0.33 Hz. In the presence of insulin, soleus muscles from SRP35TG showed a significant increase of about 90% (Mann-Whitney p < 0.05, n = 5 WT and n = 5 SRP35TG) of the maximal peak tetanic tension at the end of the fatigue protocol stimulation (Fig. 6F). Although the absolute peak tetanic tension of EDL from WT and SRP35TG mice was much lower compared to that of Soleus, the EDL from SRP35TG mice also showed a significant increase of about 100% of the peak tetanic tension at the end of the fatigue protocol (Fig. 6E). The resistance to fatigue is likely due, at least in part, to the size of the glycogen stores. Indeed, after the fatigue protocol the glycogen content of EDL (Fig. 6G) and soleus (Fig. 6H) muscles from SRP35TG mice kept on a low vitamin A diet was significantly higher than that of muscles from WT mice kept on a low vitamin A diet. It should also be pointed out that the glycogen stores of EDL and soleus muscles were smaller after the fatigue protocol (Fig. 6G and H) compared to the glycogen stores present in unstimulated muscles (Fig. 3B).

## Discussion

**Figure 7:**
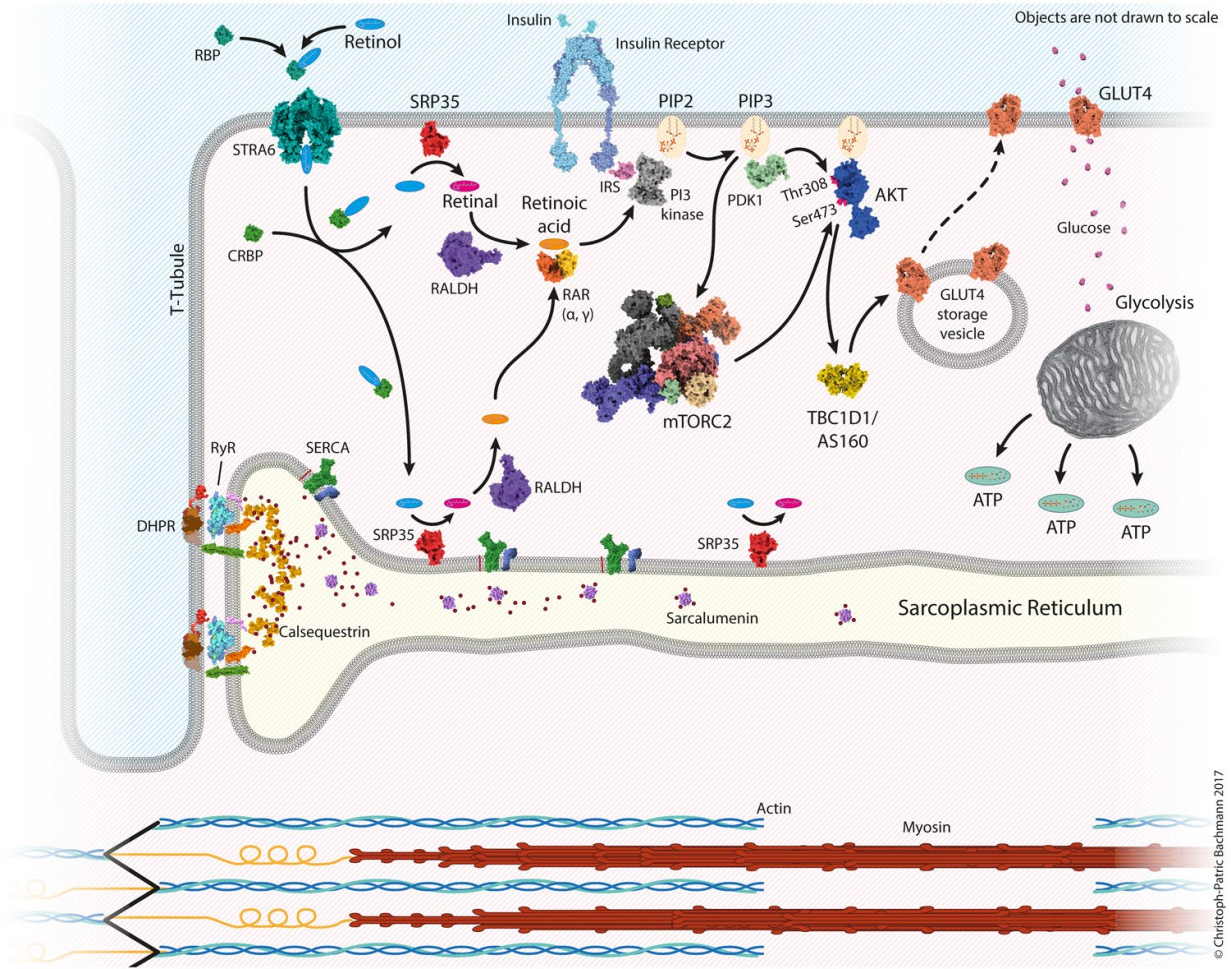
Schematic representation of the SRP-35 pathway and its effect on glucose metabolism. SRP-35 transforms retinol to retinal and the latter is converted to retinoic acid. In the presence of insulin, retinoic acid activates the Retinoic Acid Receptor α and γ (RARα and RARγ)/ Phosphoinositide 3-kinase (PI3K)/ mTOR Complex 2 (mTORC2)/Akt_S473_. Activation of Akt induces the downstream translocation of GLUT4 onto the sarcolemma leading to higher glucose uptake by muscles which in turn leads to greater glycogen stores giving the skeletal muscle machinery a higher energy source.

The present study demonstrates that the skeletal muscle protein SRP-35, a retinol dehydrogenase, is directly involved in skeletal muscle metabolism, since its over-expression results in higher glucose uptake and increased glycogen storage. In addition, SRP-35 over-expression improves muscle performance *in vivo*, an effect which occurs in the absence of changes in the ECC machinery. We also provide compelling evidence as to the mechanism by which SRP-35 leads to the increased glucose uptake in skeletal muscle. In particular we show that SRP35TG mice have an up-regulation of the signaling pathway involving Akt_S473_ phosphorylation via activation of mTORC2. Pharmacological application of atRA to intact muscles from WT mice mimics the stimulation of Akt_S473_ phosphorylation observed in SRP35TG muscles, while inhibitors of the RARα and RARγ nuclear receptors inhibit Akt_S473_ phosphorylation in WT muscle treated with pharmacological concentrations of atRA. On the basis of these results we believe that Akt_S473_ phosphorylation in skeletal muscles of SRP35TG mice is linked to the enzymatic activity of SRP-35 which produces locally within the muscle, *all*-trans-retinaldehyde, the precursor of the irreversible oxidation reaction leading to atRA formation. Altogether these data support a role for retinoic acid as the biological mediator of the metabolic response responsible for the enhanced muscle function of SRP35TG mice. Figure 7 shows a schematic representation based on the results of the present investigation, of the cellular pathways involving SRP-35 and retinoic acid metabolism in skeletal muscle.

### Overexpression of SRP-35 and atRA increase Akt_S473_ phosphorylation

Activation of Akt is due to phosphorylation of Thr308 and Ser473 by phosphinositide-dependent protein kinase 1 (PDK1) and mTORC2, respectively^31,33,38^. Overexpression of SRP-35 in skeletal muscle selectively increases the phosphorylation of the mTORC2-dependent Akt phosphorylation site Akt_S473_ and this occurred both in isolated EDL and soleus muscles from SRP35TG in the presence and absence of insulin. This implies that: (i) chronic over-expression of SRP35 in skeletal muscle increases the basal Akt_S473_ phosphorylation level, and (ii) the atRA generated by the irreversible oxidation *all*-trans-retinaldehyde, acts as a co-activator of the insulin signaling pathway leading to Akt_S473_ phosphorylation. This conclusion is also supported by a separate set of data showing that atRA mimics the effect of SRP-35 overexpression. Indeed, treatment of EDL muscles from WT mice with pharmacological concentrations of atRA enhanced insulin-induced Akt_S473_ phosphorylation levels. At variance with what was observed in EDL muscles isolated from SRP35TG mice however, incubation of EDL from WT mice with atRA in the absence of insulin did not result in Akt_S473_ phosphorylation. Our results differ from those showing that in a variety of cells types including A549, F9 and HL-60 cells, atRA alone is able to induce Akt phosphorylation^39–41^. These apparent discrepancies may be due to: (i) different experimental models, cofactors and proteins present in the cell culture medium;^42^ (ii) the lack in the EDL from WT mice of the adaptive changes induced by SRP-35 over-expression leading to an alteration of the atRA level within a subdomain of the muscle fibre. Nevertheless the present study demonstrates that atRA plays a newly identified role in the modulation of cellular functions downstream Akt_S473_ phosphorylation, including glucose metabolism.

### SRP-35 and atRA activate mTORC2 signaling

Under physiological conditions mTORC2 is the major kinase responsible for the phosphorylation of Akt_S473_^31^. In the presence of DNA damage Akt can also be phosphorylated at Ser473 by DNA-dependent protein kinase (DNA-PK)^43^. Skeletal muscle fibres from SRP35TG mice do not show evidence of nuclear damage (not shown) and thus we exclude the possibility that Akt_S473_ phosphorylation is mediated by DNA-PK. In fact our results on RImKO mice strongly support the participation of mTORC2 in the SRP-35/atRA signaling pathway. In the present study we show that atRA is unable to reverse the inhibitory effect of Rictor ablation on the phosphorylation levels of Akt_S47_ in insulin stimulated skeletal muscles^34^. This result provides unambiguous evidence that mTORC2 activation is a key component of SRP-35/atRA signaling.

### atRA: an enhancer of insulin signaling

atRA acts as an enhancer of insulin signaling since it enhances Akt_S473_ phosphorylation beyond that caused by the activation of the insulin receptor. The latter is coupled to the stimulation of PI3K activity which in turn leads to an increase of PIP_3_ on the plasma membrane, a crucial step for the recruitment of Akt onto the plasma membrane where it is phosphorylated by PDK1 and mTORC2. Akt phosphorylation mediates the metabolic effects of insulin, leading to the translocation of GLUT4 onto the plasma membrane and stimulating glucose uptake. Both atRA and SRP-35 over-expression synergize with insulin on the insulin dependent Akt_S473_ phosphorylation. The RA resulting from the oxidation of *all*-trans-retinaldehyde, the precursor of retinoic acid generated by the up-regulation of the SRP-35 dehydrogenase activity could synergize with insulin at different steps of the insulin signaling pathway. The data presented in this study provides insight as to one such possible mechanism. In particular, we found that: (i) skeletal muscles from SRP35TG mice have a 14 fold increase of the PIP_3_ content and (ii) manipulation of the myoplasmic concentration of atRA with a low Vitamin A diet affects the PIP_3_ content of skeletal muscle. Previous work has shown that inhibitors of RAR nuclear receptors down-regulate PI3K activity, the enzyme responsible for the synthesis of PIP_3_^21^. On the basis of these data and on our own results demonstrating that the Akt_S473_ phosphorylation is prevented by RARα and RARγ inhibitors, we believe that the increase of PIP_3_ content in skeletal muscle from SRP35TG results from a non-genomic effect of atRA on PI3K activity^21,44^. Accumulation of PIP_3_ in skeletal muscle membranes results in the recruitment and activation of mTORC2 kinase leading to the phosphorylation of Akt_S473_.

### SRP-35 improves muscle performance by increasing glucose uptake and glycogen store

Over-expression of SRP-35 in skeletal muscle leads to a remarkable improvement of the *in vivo* muscle performance, both in young and old (7 and 14 months old, respectively) mice. This effect could be due to a (i) gain of function of the ECC mechanism, (ii) fast to slow fibre-type switching, (iii) adaptive metabolic changes, or (iv) a combination of two or more of these mechanisms. Our results exclude the first possibility because we did not observe changes of the mechanical properties of EDL and soleus muscles. Additionally, the enhanced muscle performance is not linked to changes in the fibre type composition because there was no evidence of fibre type switching in either EDL or soleus. Both type I and type II fibres is soleus muscle from SRP35TG mice undergo a 10% increase of the minima Feret’s diameter. The increase of Akt_S473_ phosphorylation may account for this small hypertrophy^45^ of soleus muscles in SRP35TG mice and this is also consistent with the enhanced running capacity^46^. Nevertheless, such a modest hypertrophy cannot fully account for the enhanced running capacity observed in SRP35TG mice. In fact our results indicate that the enhanced muscle performance is linked to glucose metabolism. Under basal conditions, skeletal muscles from SRP35TG mice display a specific increment in GLUT4 translocation onto the sarcolemma and T tubules and this is accompanied by higher glucose uptake even in the absence of insulin. Expression and activation of GLUT4 depends on a variety of stimuli, including RA. Indeed treatment of the L6 muscle cell lines with atRA enhanced insulin-stimulated glucose uptake and increased GLUT4 expression to plasma membrane^24^. Activation of the insulin receptor stimulates PI3K activity which ultimately leads to GLUT4 translocation onto plasma membrane by the activation of two parallel signaling cascades: Akt phosphorylation and Rho-family small GTPase Rac1. Our results show that in SRP35TG mice the increased plasma membrane GLUT4 is linked to an increase of Akt_S473_ phosphorylation. The latter in turn leads to larger glycogen stores in skeletal muscle. This adaptive mechanism in SRP35TG mice is accompanied by an increase in glycogen phosphorylase (GP), the enzyme initiating the breakdown of glycogen to glucose-1-phosphate during prolonged skeletal muscle activity^7^. We are confident that the longer running distance observed in SRP35TG mice is explained by the mechanism described above.

Altogether, our results unravel a novel aspect of RA signaling in skeletal muscle and raise the possibility that SRP-35 maybe targeted to affect glucose metabolism in patients with metabolic disorders.

## Methods

### SRP-35 transgenic mice

The transgenic mouse line (generated in the Transgenic Animal Facility of Basel University) was constructed by inserting the mouse SRP-35 cDNA downstream the muscle specific creatine kinase promoter to target expression to skeletal muscle. Supplementary Fig. 1 shows a schematic representation of the construct used to create the SRP-35 over-expressing mice (SRP35TG) and a representative PCR analysis of the genomic DNA from several mouse lines generated after pronuclear injection. Mice were genotyped by PCR using genomic DNA, the following primers: 5′- GTAGCTTTTCCTGTCAATTCTGCC-3′(forward) and 5′- GAGCCCCATGGTGAAGCTT- 3′(reverse) and conditions: GoTaq G2 DNA polymerase (Promega; Madison, USA) and the following amplification protocol, 1 cycle at 95 °C for 5 min followed by 38 cycles of annealing (62 °C for 30 s), extension (72 °C for 30 s) and denaturation (95 °C for 30 s), followed by a 7 min extension cycle at 72 °C. A total of three SRP35TG mouse lines expressing the transgene to different levels were obtained. Since mouse line N°5 expressed the highest SRP-35 TG levels, the experiments described in this paper were carried out on this line and confirmed in the other two lines.

### mTORC2 KO (RImKO) mice

3 months old male muscle-specific rictor knockout mice (RImKO) were obtained from Prof. M. Rüegg’s and Prof. M. Hall’s laboratories^34^.

### Special diet

WT and SRP35TG mice were fed a Low Vitamin A Diet (LVAD; 4 UI/g; Scientific Animal food and Engineering) for 2 generations. The mice of the second generation were sacrificed at 4 months of age^37^.

### *In vivo* and *in vitro* assessment of muscle function

*In vivo* muscle function was assessed using the voluntary running wheel on 7 and 14 months old male mice as previously described^47^. For *in vitro* assessment of muscle function, force measurements were assessed using a force transducer (MyoTonic, Heidelberg), and by measuring the force generated in response to different protocols including a single twitch (15 V pulses for 0.5 ms duration) and tetanic frequency pulses (50, 100 and 150 Hz) in soleus (slow muscle) and (50, 100 and 200 Hz) in *extensor digitorum longus* (EDL, fast muscle). Resistance of muscle to fatigue was also measured in EDL and Soleus muscles isolated from WT and SRP35TG mice kept for 2 generations on a low Vitamin A diet. EDL and Soleus were incubated in Krebs Ringer containing 100 nM insulin, and stimulated with a train of tetanic stimulation (EDL: 70 Hz, 300 ms duration; Soleus: 600 ms duration, 50 Hz) delivered at 0.33 Hz^48^

### Basic metabolic rate

The metabolic rate of WT and TG (7 months old) male mice was assessed by indirect calorimetry using a Comprehensive Lab Animal Monitoring System (Oxymax/CLAMS; Columbus Instruments, Columbus, OH, USA). Following an initial 48 h acclimatization, mice were monitored every 17 min for 24 h for a complete 12 h active (dark) and 12 h inactive (light) cycle. Oxygen consumption (VO_2_), CO_2_ production (VCO_2_), physical activity, heat production and respiratory exchange ratio (RER = V˙CO_2_ /VO_2_) were measured during 72 hours.

### Histological examination, changes in muscle fiber type and glycogen content

Calculation of the minimal Feret’s diameter, the closest possible distance between the two parallel tangents of an object, was determined as previously described^49^. Images were obtained using an Olympus IX series microscope and analyzed using the CellP Software. The fiber type composition was assessed by determining the expression of different myosin heavy chain isoforms by high resolution gel electrophoresis followed by Coomassie Brilliant blue gel staining as described^50,51^.

Glycogen content was assessed enzymatically on snap frozen EDL and soleus muscles using a hexokinase-dependent, kit according to the manufacturer’s instructions (GAHK-20, Sigma-Aldrich; USA). A control to measure free glucose levels in the muscle (not related to glycogen stores), was also performed.

### Gel electrophoresis and western blotting

Total homogenates, sarcoplasmic reticulum (SR) and skeletal muscle subcellular fractions longitudinal sarcoplasmic reticulum (LSR) and terminal cisternae (TC), were prepared as described^27^. Protein concentration was determined using a kit from BioRad (Bio-Rad, 500–0006) using bovine serum albumin as standard; proteins were separated on SDS-PAG gels, transferred onto nitrocellulose membranes and probed with commercially available antibodies. Immunopositive bands were visualized by chemiluminescence.

### Signaling pathways involved in glucose uptake: Akt and AMPK phosphorylation

Isolated soleus muscles from 28 weeks old mice were used to determine the levels of Akt and AMPK phosphorylation. Muscles were homogenized in lysis buffer (10% Glycerol, 5% β-mercaptoethanol, 2.3% SDS, 62.5 mM Tris-HCl pH 6.8 and 6 M urea, supplemented with 1% phosphatase inhibitor cocktails 2 and 3 Sigma Aldrich) at a concentration of 10 mg of muscle/ml buffer. Subsequently, 50 µg of protein were separated on a 10% SDS PAG, transferred onto nitrocellulose (Amersham), and probed with the following primary antibodies: phospho-AMPK (Thr172), AMPK alpha, phospho-Akt (ser 473), phopho-Akt (thr 308) and Akt total from Cell Signaling and Desmin (used as housekeeping loading control) from Santa Cruz. The intensity of the immunopositive bands was determined using Image J.

### Glucose Uptake

Glucose uptake by isolated EDL and soleus muscles was measured in isolated muscles incubated for 1 h at room temperature in Krebs-Ringer buffer supplemented with 1 mM glucose, 10 mM sodium pyruvate and 0.5% bovine serum albumin. The medium was continuously gassed with 95% O_2_ and 5% CO_2_. For electrical stimulation, muscles were stimulated with a 300 ms train of action potentials of 0.5 ms duration at a frequency of 80 Hz by using a stimulator (Myotronic, Heidelberg Germany); the 300 ms trains of action potentials were delivered for a 5 min at a frequency of 0.27 Hz. For insulin stimulation, muscles were incubated in the presence or absence of insulin (100 nM; Novo Nordisk Pharma AG; Bagsværd, Denmark) for 10 min at room temperature. Contralateral non-stimulated muscles were used as controls. Stimulated or control muscles were then incubated with 0.375 µCi/ml Deoxy-D-glucose, 2-[1,2–^3^H (N)] (2-[^3^H]-DG (Perkin Elmer, Waltham, MA) for an additional 20 min at room temperature, flash frozen and stored in liquid nitrogen until ready for use. The amount of radioactive glucose taken up by the muscles was assessed by liquid scintillation counting, using a Packard 1900 TR liquid scintillation analyzer.

### Glucose and insulin tolerance tests

For the glucose tolerance test (GTT), following a 6 h fast and starting in the morning, mice were injected intraperitoneally with a glucose-containing solution (1 µg glucose/g of body weight). Glucose plasma levels were determined at different time points (0, 15, 30, 60 and 90 min) using a glucometer (Freestyle; Abbott Diabetes Care Inc., Alameda, CA; USA). Insulin plasma levels were determined at different time points (0, 15 and 30 min) using an insulin ELISA kit (Mercodia; Uppsala, Sweden) following the manufacturer’s recommendations.

### Effect of all-trans-retinoic acid (atRA)

EDL and soleus muscles from 28 weeks old WT mice, were cleaned in a Ringer solution continuously gassed with 95% O_2_ and 5% CO_2_ at room temperature. The muscles were incubated in the presence or absence of 10 µM All-trans-retinoic acid (atRA, dissolved in DMSO; Sigma), control muscle were incubated with the vehicle DMSO. After 30 or 60 min of incubation, the muscles were washed and stored in liquid N_2_. In some experiments insulin (100 nM) was also added to the muscles incubated with atRA and the controls. The muscles were homogenized; proteins separated by SDS/PAGE and transferred to a nitrocellulose membranes. Phosphorylation sites and total protein content of AMPK and AKT were analyzed. All experiments were performed under red light illumination.

### Effect of Retinoic acid Receptor (RaR) inhibitors

EDL and soleus muscles isolated from WT mice were incubated in Ringer’s solution containing one of 3 different RAR inhibitors (RARα, RARβ or RARγ inhibitors), 100 nM insulin and in presence or absence of 10 µM atRA. Muscles from the contralateral leg were taken as controls and incubated with the same RAR inhibitor as their counterpart muscles, together with 100 nM insulin and DMSO. After a 30 min incubation with the RARα inhibitor Ro 41–5253 (50 nM; Sigma), RARβ inhibitor LE 135 (220 nM; Sigma) or RARϒ inhibitor Acacetin (30 uM; Sigma), insulin and atRA/DMSO, the muscles were flash frozen in liquid N_2_ and subsequently analysed by western blotting for Akt and AMPK phosphorylation. All experiments were performed under red light illumination.

### PIP_3_ measurements

PIP_3_ levels were measured in muscles of WT and SRP35TG mice fed a standard chow or a low vitamin A diet. Freshly isolated EDL muscles were flash frozen in liquid N_2_ and ground using a pestle and mortar. Lipids were extracted from the powdered muscles and PIP_3_ content was assessed as previously described^51^ using the PIP_3_ Mass ELISA kit (K-2500s, Echelon, USA).

### Animal permits

All experiments were conducted according to the Swiss Veterinary Law and institutional guidelines and were approved by the Swiss authorities (Kantonal permits 1728 and 2115). All animals were housed in a temperature-controlled room with a 12 h light–12 h dark cycle and had free access to food and water.

### Statistical analysis

Statistical analysis was performed using the software OriginPro 8.6.0 (OriginLab Corporation). Comparisons of two groups were performed using the Student’s *t*-test, for groups of three or more comparisons were made using the ANOVA test followed by the Bonferroni post-hoc test unless otherwise stated. Means were considered statistically significant when P values were < 0.05. All figures were created using Adobe Photoshop CS6 or R Studio (version 0.99.891 or newer).

## Electronic supplementary material

Supplementary Information

## References

[1] Loiselle DS, Johnston CM, Han JC, Nielsen PM, Taberner AJ (2016). Muscle heat: a window into the thermodynamics of a molecular machine. Am. J. Physiol. Heart Circ. Physiol..

[2] Sopariwala DH (1985). Sarcolipin overexpression improves muscle energetics and reduces fatigue. J. Appl. Physiol..

[3] Bottinelli R, Reggiani C (2000). Human skeletal muscle fibres: molecular and functional diversity. Prog. Biophys. Mol. Biol..

[4] Schiaffino S, Reggiani C (2011). Fiber types in mammalian skeletal muscles. Physiol. Rev..

[5] Calderón JC, Bolaños P, Caputo C (2010). Myosin heavy chain isoform composition and Ca^2+^ transients in fibres from enzymatically dissociated murine soleus and extensor digitorum longus muscles. J. Physiol..

[6] Chemello F (2011). Microgenomic Analysis in Skeletal Muscle: Expression Signatures of Individual Fast and Slow Myofibers. PLoS One.

[7] Westerblad H, Bruton J, Katz A (2010). Skeletal muscle: energy metabolism, fiber types, fatigue and adaptability. Exp Cell Res..

[8] Zurlo F, Larson K, Bogardus C, Ravussin E (1990). Skeletal muscle metabolism is a major determinant of resting energy expenditure. J. Clin. Invest..

[9] Jensen J, Rustad P, Kolnes AJ, Lai YC (2011). The role of skeletal muscle glycogen breakdown for regulation of insulin sensitivity by exercise. Front. Physiol..

[10] Douen AG (1990). Exercise induces recruitment of the “insulin-responsive glucose transporter”. Evidence for distinct intracellular insulin- and exercise-recruitable transporter pools in skeletal muscle. J. Biol. Chem..

[11] Mueckler M (2001). Insulin resistance and the disruption of GLUT4 trafficking in skeletal muscle. J. Clin. Invest..

[12] Hansen PA, Wang W, Marshall BA, Holloszy JO, Mueckler M (1998). Dissociation of GLUT4 translocation and insulin-stimulated glucose transport in transgenic mice overexpressing GLUT1 in skeletal muscle. J. Biol. Chem..

[13] Klip A, Sun Y, Chiu TT, Foley KP (2014). Signal transduction meets vesicle traffic: the software and hardware of GLUT4 translocation. Am. J. Physiol. Cell Physiol..

[14] Treves S (2009). Minor sarcoplasmic reticulum membrane components that modulate excitation-contraction coupling in striated muscles. J. Physiol..

[15] Duester G (1996). Involvement of alcohol dehydrogenase, short-chain dehydrogenase/reductase, aldehyde dehydrogenase, and cytochrome P450 in the control of retinoid signaling by activation of retinoic acid synthesis. Biochemistry.

[16] Persson B (2009). The SDR (short-chain dehydrogenase/reductase and related enzymes) nomenclature initiative. Chem. Biol. Interact..

[17] Treves S (2012). SRP-35, a newly identified protein of the skeletal muscle sarcoplasmic reticulum, is a retinol dehydrogenase. Biochem. J..

[18] Duong V, Rochette-Egly C (2011). The molecular physiology of nuclear retinoic acid receptors. From health to disease. Biochim. Biophys. Acta.

[19] Cunningham T, Duester G (2015). Mechanisms of retinoic acid signalling and its roles in organ and limb development. Nat. Rev. Mol. Cell Biol..

[20] Masia´ S, Alvarez S, de Lera AR, Barettino D (2007). Rapid, nongenomic actions of retinoic acid on phosphatidylinositol-3-kinase signaling pathway mediated by the retinoic acid receptor. Mol. Endocrinol..

[21] Berry DC, Noy N (2009). All-trans-retinoic acid represses obesity and insulin resistance by activating both peroxisome proliferation-activated receptor beta/delta and retinoic acid receptor. Mol. Cell Biol..

[22] Sugita S (2011). Increased Systemic Glucose Tolerance with Increased Muscle Glucose Uptake in Transgenic Mice Overexpressing RXRγ in Skeletal Muscle. PLoS One..

[23] Lee Y (2008). Retinoic acid leads to cytoskeletal rearrangement through AMPK-Rac1 and stimulates glucose uptake through AMPK-p38 MAPK in skeletal muscle cells. J. Biol. Chem..

[24] Sleeman M, Zhou H, Rogers S, Wah K, Best J (1995). Retinoic acid stimulates glucose transporter expression in L6 muscle cells. Molec. Cell Endocrinol..

[25] Parolin M (1999). Regulation of skeletal muscle glycogen phosphorylase and PDH during maximal intermittent exercise. Am. J. Physiol..

[26] Dos Santos JM, Benite-Ribeiro SA, Queiroz G, Duarte JA (2012). The effect of age on glucose uptake and GLUT1 and GLUT4 expression in rat skeletal muscle. Cell Biochem. Funct..

[27] Saito A, Seiler S, Chu A, Fleischer. S (1984). Preparation and morphology of sarcoplasmic reticulum terminal cisternae from rabbit skeletal muscle. J. Cell Biol..

[28] Gowans GJ, Hardie DG (2014). AMPK: a cellular energy sensor primarily regulated by AMP. Biochem. Soc. Trans..

[29] Muindi J, Frankel SR, Huselton C (1992). Clinical pharmacology of oral all-trans retinoic acid in patients with acute promyelocytic leukemia. Cancer Res..

[30] Mukherjee R (1997). Sensitization of diabetic and obese mice to insulin by retinoid X receptor agonists. Nature.

[31] Sarbassov DD, Guertin DA, Ali SM, Sabatini DM (2005). Phosphorylation and regulation of Akt/PKB by the rictor-mTOR complex. Science.

[32] Laplante M, Sabatini. D (2009). mTOR signaling at a glance. J. Cell Sci..

[33] Liko D, Hall MN (2015). mTOR in health and in sickness. J. Mol. Med..

[34] Bentzinger F (2008). Skeletal muscle-specific ablation of raptor, but not of rictor, causes metabolic changes and results in muscle dystrophy. Cell Metab..

[35] Liu P (2015). PtdIns(3,4,5)P3-dependent activation of the mTORC2 kinase complex. Cancer Discov..

[36] López-Carballo G, Moreno L, Masiá S, Pérez P, Barettino D (2002). Activation of the phosphatidylinositol 3-kinase/Akt signaling pathway by retinoic acid is required for neural differentiation of SH-SY5Y human neuroblastoma cells. J. Biol. Chem..

[37] Obrochta KM, Kane MA, Napoli JL (2014). Effects of diet and strain on mouse serum and tissue retinoid concentrations. PLoS One.

[38] Laplante M, Sabatini D (2012). mTOR signaling in growth control and disease. Cell.

[39] García-Regalado A, Vargas M, García-Carrancá A, Aréchaga-Ocampo E (2013). & González-De la Rosa, C. Activation of Akt pathway by transcription-independent mechanisms of retinoic acid promotes survival and invasion in lung cancer cells. Mol. Cancer..

[40] Lee Y, Lee JY, Kim MH (2014). PI3K/Akt pathway regulates retinoic acid-induced Hox gene expression in F9 cells. Develop. Growth Differ..

[41] Matkovic K, Brugnoli F, Bertagnolo V, Banfic H, Visnjic D (2006). The role of the nuclear Akt activation and Akt inhibitors in all-trans-retinoic acid-differentiated HL-60 cells. Leukemia.

[42] Luo X, Ross C (2005). Physiological and receptor-selective retinoids modulate interferon γ signaling by increasing the expression, nuclear localization, and functional activity of interferon regulatory factor-1. J. Biol. Chem..

[43] Bozulic L, Surucu B, Hynx D, Hemmings BA (2008). PKBalpha/Akt1 acts downstream of DNA-PK in the DNA double-strand break response and promotes survival. Mol. Cell.

[44] Qiaoa J (2012). PI3K/AKT and ERK regulate retinoic acid-induced neuroblastoma cellular differentiation. Biochem. Biophys. Res. Commun..

[45] Lai KM (2004). Conditional activation of akt in adult skeletal muscle induces rapid hypertrophy. Mol, Cell Biol..

[46] Keni OJ, Loennechen JP, Wisløff U, Ellingsen Ø (2002). Intensity-controlled treadmill running in mice: cardiac and skeletal muscle hypertrophy. J. Appl. Physiol..

[47] Delbono O (2007). Loss of skeletal muscle strength by ablation of the sarcoplasmic reticulum protein JP-45. Proc. Natl. Acad. Sci. USA.

[48] Johansson C, Lunde PK, Gothe S, Lannergren J, Westerblad H (2003). Isometric force and endurance in skeletal muscle of mice devoid of all known thyroid hormone receptors. J. Physiol..

[49] Briguet A, Courdier-Fruh I, Foster M, Meier T (2004). & Magyar, J.P. Histological parameters for the quantitative assessment of muscular dystrophy in the mdx-mouse. Neuromuscul Disord..

[50] Talmadge RJ, Roy RR (1993). Electrophoretic separation of rat skeletal muscle myosin heavy chain isoforms. J. Appl. Physiol..

[51] Bachmann C (2017). Cellular, biochemical and molecular changes in muscles from patients with X-linked myotubular myopathy due to MTM1 mutations. Hum. Mol. Genet..

