# Supplementary material for: Over-expression of a retinol dehydrogenase (SRP35/DHRS7C) in skeletal muscle activates mTORC2, enhances glucose metabolism and muscle performance
Source: Sci Rep. 2018 Jan 12;8:636. doi: 10.1038/s41598-017-18844-3 (PMC5766524; doi:10.1038/s41598-017-18844-3)

Supplementary information to the manuscript entitled: "Over-expression of a retinol dehydrogenase (SRP35/DHRS7C) in skeletal muscle activates mTORC2, enhances glucose metabolism and muscle performance",

by Alexis Ruiz, Erez Dror, Christoph Handschin Regula Furrer, Joaquin Perez-Schindler, Christoph Bachmann, Susan Treves and Francesco Zorzato

<u>Supplementary Table 1:</u> Analysis of the contractile properties of EDL and soleus muscles from WT and SRP35TG mice.

		EDL		Soleus	
		WT	TG	WT	TG
Twitch					
	Half time to peak (ms)	3.4 ± 0.2	3.4 ± 0.4	5.1 ± 0.2	5.45 ± 0.5
	Time to Peak (ms)	11.8 ± 1.04	12.7 ± 1.9	21.0 ± 1.1	22.62 ± 1.8
	Half Relaxation time (ms)	14.0 ± 2.3	15.7 ± 3.3	28.9 ± 3.1	34.08 ± 6.4
	Force (mN/mm^2)	99.69 ± 15.3	95.8 ± 18.7	48.7 ± 11.1	45.78 ± 16.6
Tetani					
50 Hz	Half Relaxation time (ms)	13.6 ± 3.4	15.1 ± 3.6	42.9 ± 3.5	43.9 ± 3.4
	Force (mN/mm^2)	224.1 ± 52.1	246.4 ± 47.6	202.9 ± 32.4	204.7 ± 47.4
100 Hz	Half Relaxation time (ms)	19.8 ± 2.6	20.2 ± 2.8	48.6 ± 3.4	51.5 ± 3.6
	Force (mN/mm^2)	387 ± 61.0	405.3 ± 67.6	242 ± 27	243.8 ± 44.2
150 Hz Soleus<br>200 Hz EDL	Half Relaxation time (ms)	26.11 ± 2.0	28.3 ± 7.9	53.6 ± 3.5	55.3 ± 4.2
	Force (mN/mm^2)	486.3 ± 36.6	480.4 ± 75.9	244.2 ± 29.9	242.3 ± 46.8
Muscle wet weight		13.8 ± 1.4	13.4 ± 1.5	11.0 ± 2.0	12.3 ± 12.3
Muscle lenght (mm)		13.4 ± 0.2	13.5 ± 0.8	12.7 ± 0.4	13.0 ± 0.5

Data were recorded from EDL and soleus muscles isolated from 7 months old mice. Force records and analysis were performed using Chart5 (ADInstruments, Dunedin, New Zealand). Values represent the mean ±S.D, statistical analysis using the Student's *t* test showed no significant differences between WT (n=7) and SRP35TG (n=13) mice.

<u>Supplementary Table 2</u>: List of primary antibodies and suppliers used for western blot analysis.

Antibody Target	Company	Serial N°	Working Dilution	Final Concentration	Molecular Weight
Akt (C67E7)	Cellsignaling	4691S	1:500	I.N.A	60 KDa
Akt(ser473)	Cellsignaling	4058S	1:500	I.N.A	60 KDa
Akt(Thr308)	Cellsignaling	9275S	1:500	I.N.A	60 KDa
Albumin	Bethyl	A90-134P	1:80000	I.N.A	66 KDa
AMPKα tot	Cellsignaling	2532S	1:500	I.N.A	60 KDa
AMPK(Thr172)	Cellsignaling	2531S	1:500	I.N.A	62 KDa
Ca_V 1.1	Santa Cruz	sc-8160	1:250	0.8 ug/ml	170 KDa
Calsecuestrin-1 (CS)	Sigma	CO743	1:200	0,9 ug/ml	60 KDa
Desmin (H-76)	Santa Cruz	SC-14026	1:500	0.4 ug/ml	53 KDa
Dihidripiridine receptor (β1a)	Santa Cruz	SC-32079	1:250	0.8 ug/ml	170 KDa
JP-45	Made in our lab	Zorzato et al 2000.	1:5000	0.66 ug/ml	45 KDa
Glut1	Santa Cruz	SC- 377228	1:100	200 ug/ml	45 KDa
Glut4	abcam	Ab654	1:2000	I.N.A	45 KDa
Glycogen phosphorylase (GP)	Santa cruz	Sc-46347	1:500	1 ug/ml	97 KDa
Rictor	Cellsignaling	2140S	1:500	I.N.A	200 KDa
RyR1	Thermo Scientific	MA3-916	1:5000	I.N.A	565 KDa
Sarcalumenin	Thermo Scientific	MA3-932	1:1100	I.N.A	53/160 KDa
Serca 1	Santa Cruz	SC-8093	1:5000	0.04 µg/ml	110 KDa
Serca 2	Santa Cruz	Sc-8095	1:5000	0.04 µg/ml	110 KDa
SGK (G-4)	Santa Cruz	SC-377360	1:500	0.4 µg/ml	49/60 KDa
SGK (H-4)	Santa Cruz	SC-28338	1:500	0.4 µg/ml	49/60 KDa
SRP35	Sigma	SAB1307061	1:1000	0.5 µg/ml	35 KDa

I.N.A: information not available

Supplementary Figure 1: Creation of an SRP-35 transgenic mouse model. (A) Schematic representation outlining the strategy used to create the SRP-35 over-expressing mouse model. The cDNA encoding mouse SRP-35 was inserted downstream the muscle specific creatine kinase promoter to target expression to skeletal muscle. A polyadenylation sequence derived from SV40 (SV40 poly A) was inserted at the 3'end of the transcript. The bottom portion shows an agarose gel electrophoresis showing two positive lanes (arrows, lanes 1 and 15) after amplification of genomic DNA obtained from transgenic mice. **(B)** PCR analysis of the genomic DNA from WT and SRP35TG mice. Genomic DNA was amplified using GoTaq G2 DNA polymerase with the primers 5` GTAGCTTTTCCTGTCAATTCTGCC 3`(forward) and 5` GAGCCCCATGGTGAAGCTT 3`(reverse). A positive band was only obtained in the lanes labeled SRP35TG and not Wild type. **(C)** Coomassie blue staining of total sarcoplasmic reticulum proteins isolated from muscles dissected from WT and SRP35TG mice. Ten micrograms of protein were loaded per lane and separated on a 7.5% SDS/PAGE. The asterisk shows the presence of the SRP-35 protein in the total SR muscle samples from SRP35TG mice. **(D)** Western blot analysis of the SRP-35 protein content in muscles from 3 different mouse families (family 4, 5 and 6) over-expressing SRP-35 in their skeletal muscles. The transgenic mice belonging to families 4, 5 and 6 showed an increase of SRP-35 expression of 28%, 61% and 23%, respectively compare to their WT littermates.

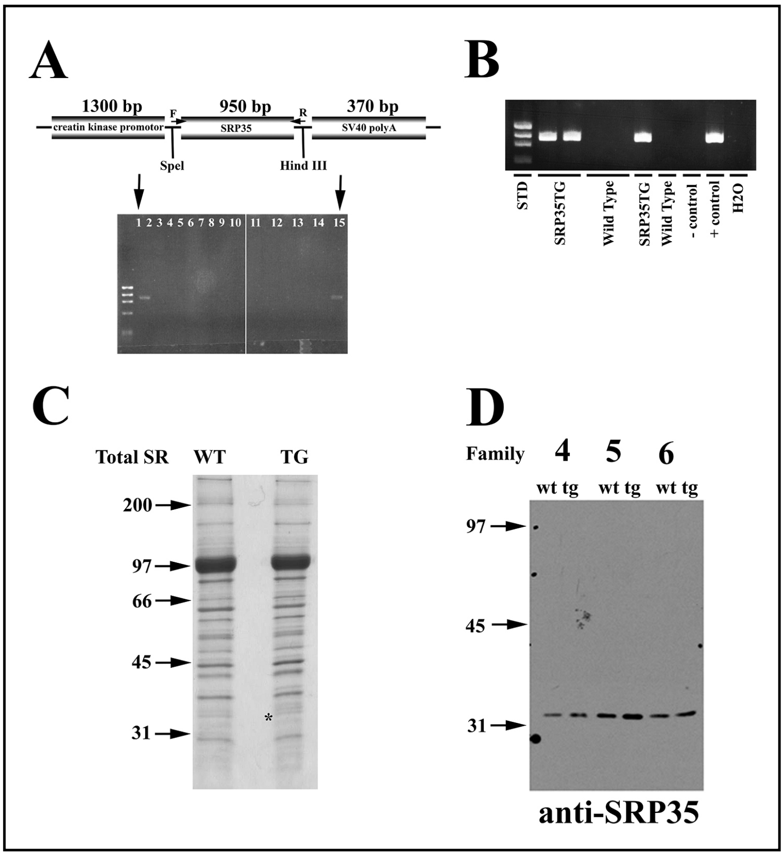

<u>Supplementary Figure 2</u>: **The protein content of the glucose transporter 1 (GLUT1) is not affected by SRP-35 over-expression and atRA treatment does not influence the protein content of AMPK and Akt. (A)** Western blot analysis of the skeletal muscle t-tubule sarcolemmal fraction (R1) fraction isolated from WT and SRP35TG mice (pooled fractions from 7 mice). Thirty micrograms of protein were loaded per lane, separated on 10% SDS/PAGE and blotted onto nitrocellulose. Blots show that the content of GLUT1 was similar in WT and SRP35TG muscles. **(B)** Western blot of a total muscle homogenate from EDL muscles isolated from WT and SRP35TG mice. Fifty micrograms of protein were loaded per lane, separated on a 10 % SDS/PAGE, blotted onto nitrocellulose, and probed with the anti-GLUT1 antibody. The content of GLUT1 was similar in total homogenates from WT and TG mouse EDLs. **(C)** Bar histogram of total Akt and AMPK content as determined by western blot analysis of EDL muscles from WT mice treated with 10 µM atRA for 30 and 60 min. The signal from the immunopositive band from the EDL treated with atRA was compared to that obtained from the contralateral control EDL muscle treated with DMSO, which was considered 100% (empty bar). Data are presented as % of control (±S.D.). Experiments were performed on EDL from 7 mice. No significant differences (Student's *t* test) in the protein content of Akt and AMPK were found.

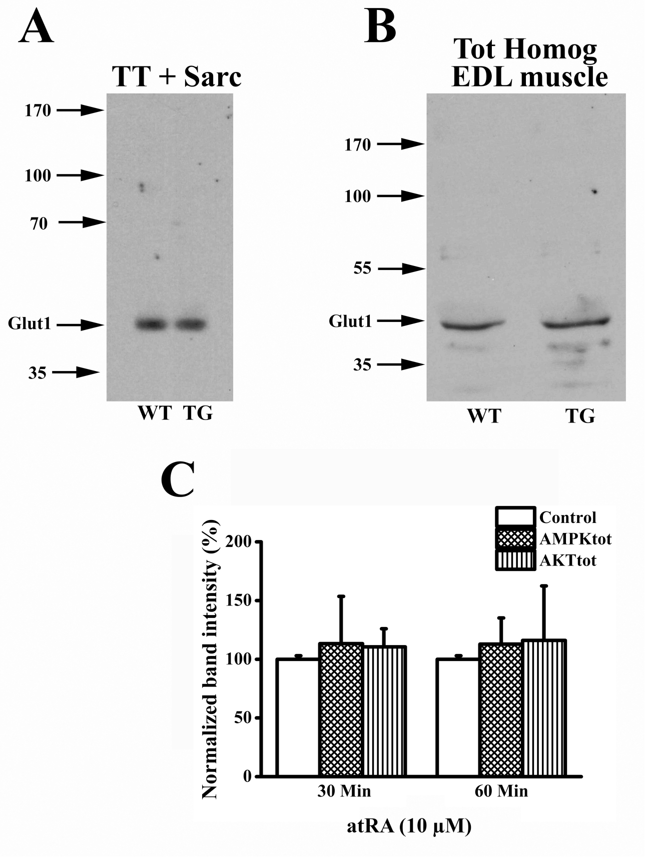

<u>Supplementary Figure 3</u>: Specificity of the antibodies used in the present investigation. Representative western blots showing the immunopositive bands on total muscle homogenates. Fifty micrograms of protein were separated on 10% SDS/PAGE, blotted onto nitrocellulose and probed using the indicated antibodies. The monoclonal anti- Akt and AMPK antibodies were incubated overnight, followed by washing and probing using a peroxidase-conjugated secondary anti mouse antibody. The immunopositive signal was visualized using an enhanced chemiluminescence kit.

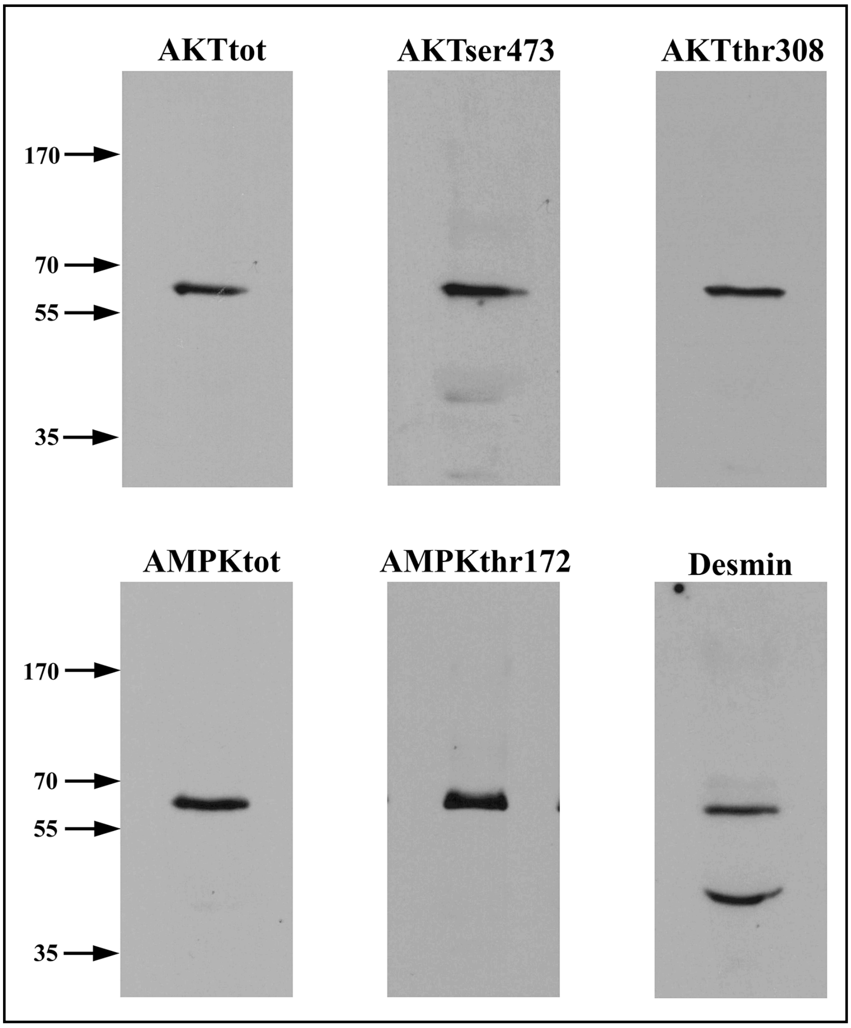

Supplement: Supplementary file 1 — Supplementary Information [file 41598_2017_18844_MOESM1_ESM.pdf]
